# Tubular intestinal duplication extending from the stomach to the ileum associated with multiple intestinal atresia and situs inversus: a case report

**DOI:** 10.1186/s40792-023-01728-2

**Published:** 2023-08-09

**Authors:** Naruki Higashidate, Saki Sakamoto, Nobuyuki Saikusa, Yoshinori Koga, Daisuke Masui, Hirotomo Nakahara, Misa Nakamura, Mamoru Saikusa, Masahiro Kinoshita, Tatsuru Kaji

**Affiliations:** 1 Department of Pediatric Surgery, Kurume University School of Medicine, 67 Asahi-machi, Kurume, Fukuoka 830-0011 Japan; 2 Department of Pediatrics and Child Health, Kurume University School of Medicine, 67 Asahi-machi, Kurume, Fukuoka 830-0011 Japan

**Keywords:** Intestinal duplication, Visceral inversion, Intestinal atresia, Stapled anastomosis, Case report

## Abstract

**Background:**

Duplication of the alimentary tract can occur in any of its parts. For duodenal duplication, complete resection is particularly difficult when the ampulla of Vater is on the duplicated lumen and a deliberate management is necessary.

**Case presentation:**

A 0-day female baby was referred to our department due to abdominal distention. The X-ray examination showed dextrocardia and opacity of the liver on the left side and abdominal ultrasonogram revealed remarkable intestinal dilatation. Therefore, urgent laparotomy was performed on the day of birth. Complete situs inversus of the abdominal organs was revealed, and the origin of the jejunum was on the left side and was accompanied by tubular intestinal duplication. The origin of the duplicated intestine was at the pancreatic head’s dorsal area. There were two points of type Шa atresia on the ileum. Therefore, we spared the duplicated intestine with a length of 3 cm to secure the passage of the biliary and pancreatic juices by a functional-side-to-side anastomosis with a 45-mm Endo-GIA™ camel load (Medtronic, Minneapolis, MN, USA). The ileum was transected at the distal side of the atresia point, and end-to-end jejunoileostomy was performed. Postoperative gastrointestinal series revealed remnants of the duplicated alimentary tract on the dorsal area of the stomach.

**Conclusions:**

Identifying the position of the ampulla of Vater is crucial in the surgery of alimentary tract duplication with duodenal involvement. However, in the present case, it was difficult to identify whether the ampulla of Vater was on the true or duplicated lumen, and we had to spare the duplicated duodenum. Stapler anastomosis could be performed safely even in neonatal cases.

## Background

Duplication of the alimentary tract can occur at any point of the alimentary tract, from the mouth to the anus, with varying known gastrointestinal anomalies [1]. For duodenal duplication, identifying the position of the ampulla of Vater is crucial, because complete resection is particularly difficult when it is opened to the duplicated lumen [2]. Herein, we report a case of tubular intestinal duplication extending from the stomach to the ileum associated with multiple intestinal atresia and situs inversus, which was treated by stapled side-to-side anastomosis. This manuscript was prepared following the CARE guidelines.

## Case presentation

**Fig. 1:**
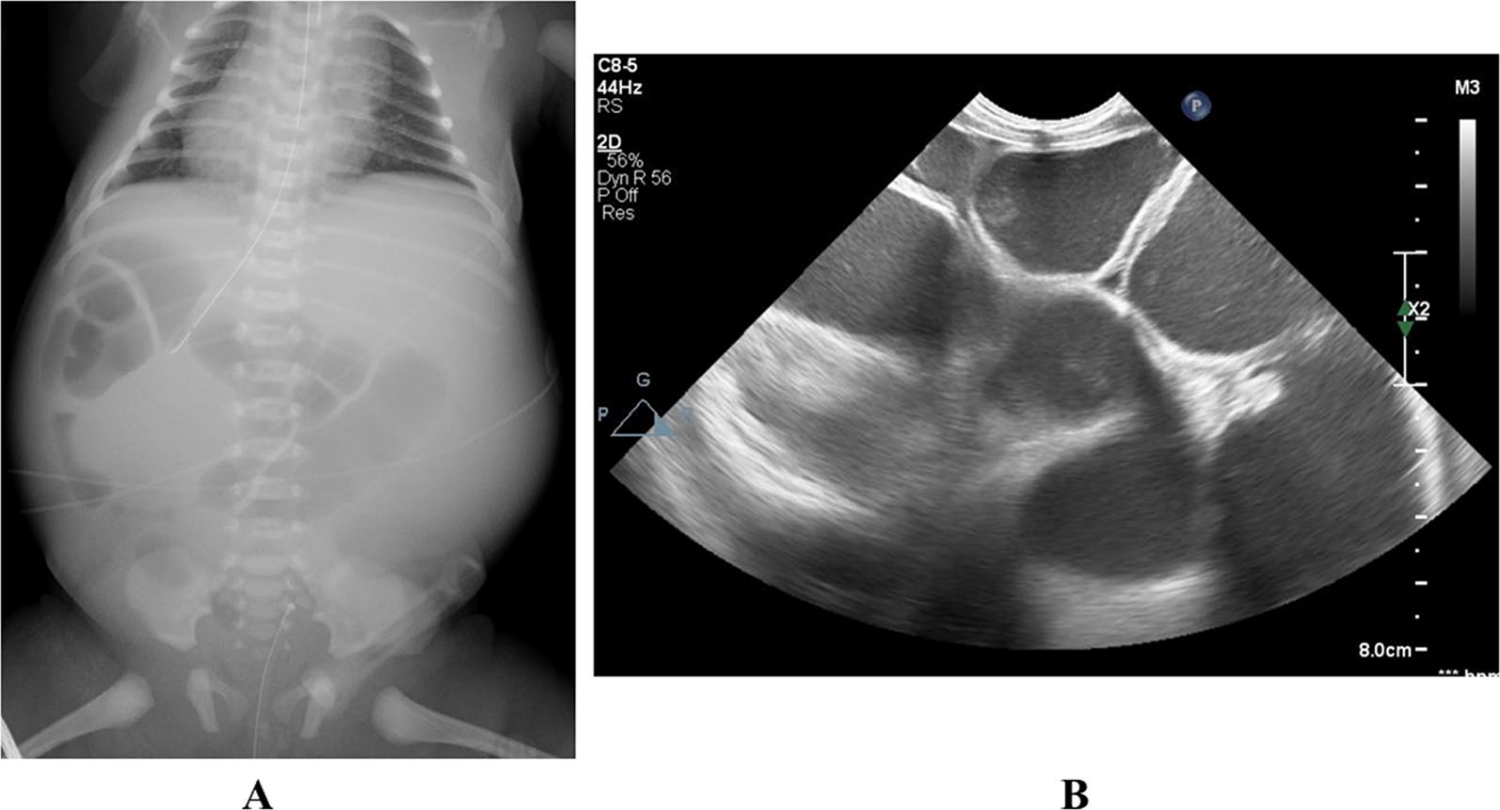
Preoperative imaging studies**. A** X-ray film revealed dextrocardia, opacity of the liver on the left side, and shifting of the nasogastric tube to the right side. **B** Abdominal ultrasonogram showed remarkable dilatation of the intestine

**Fig. 2:**
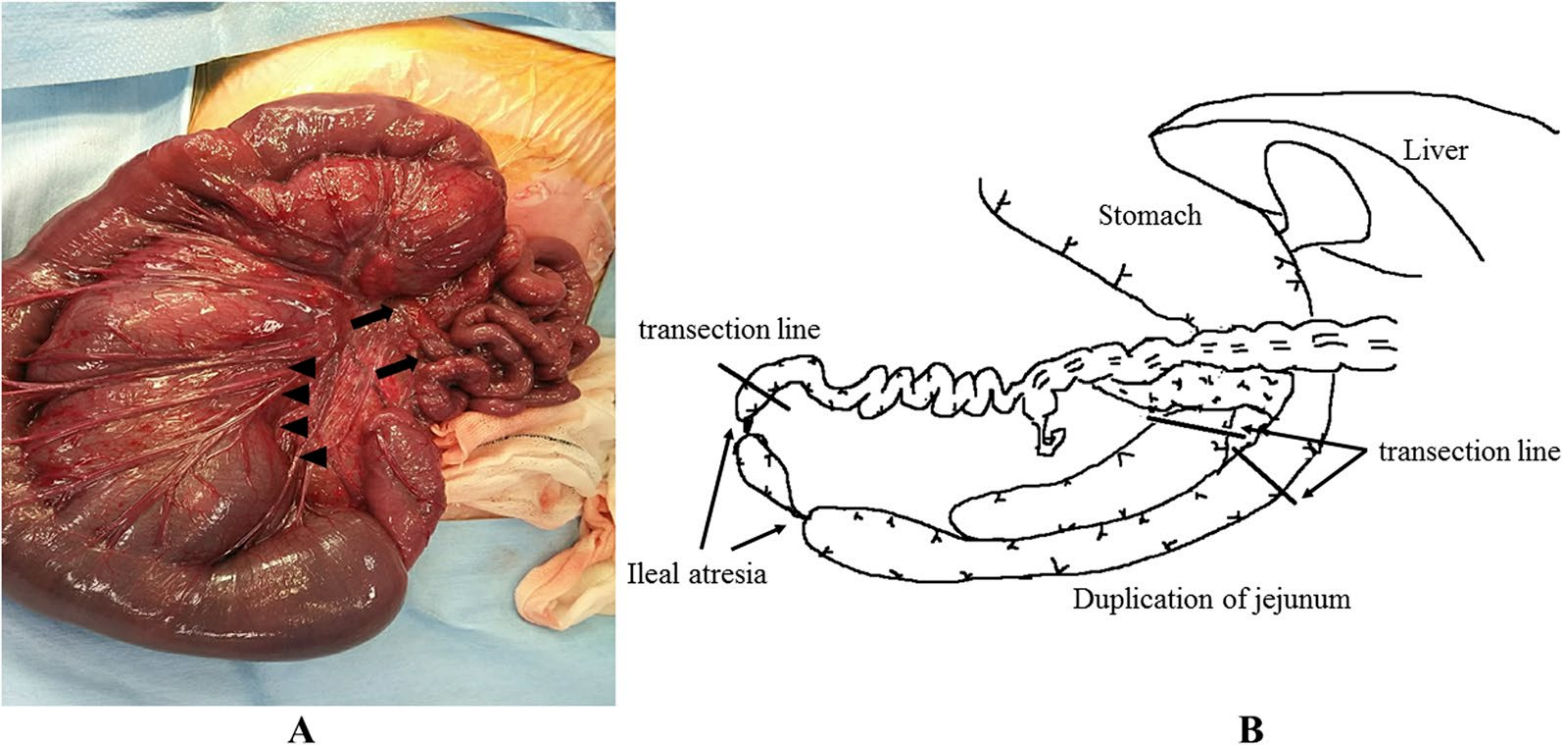
Intraoperative findings. **A** The tubular intestinal duplication was found on the mesenteric side with a length of 40 cm (arrow head). Two points of type Шa ileal atresia at intervals of 5 cm were also found (arrow). **B** Schema of the intraoperative findings

**Fig. 3:**
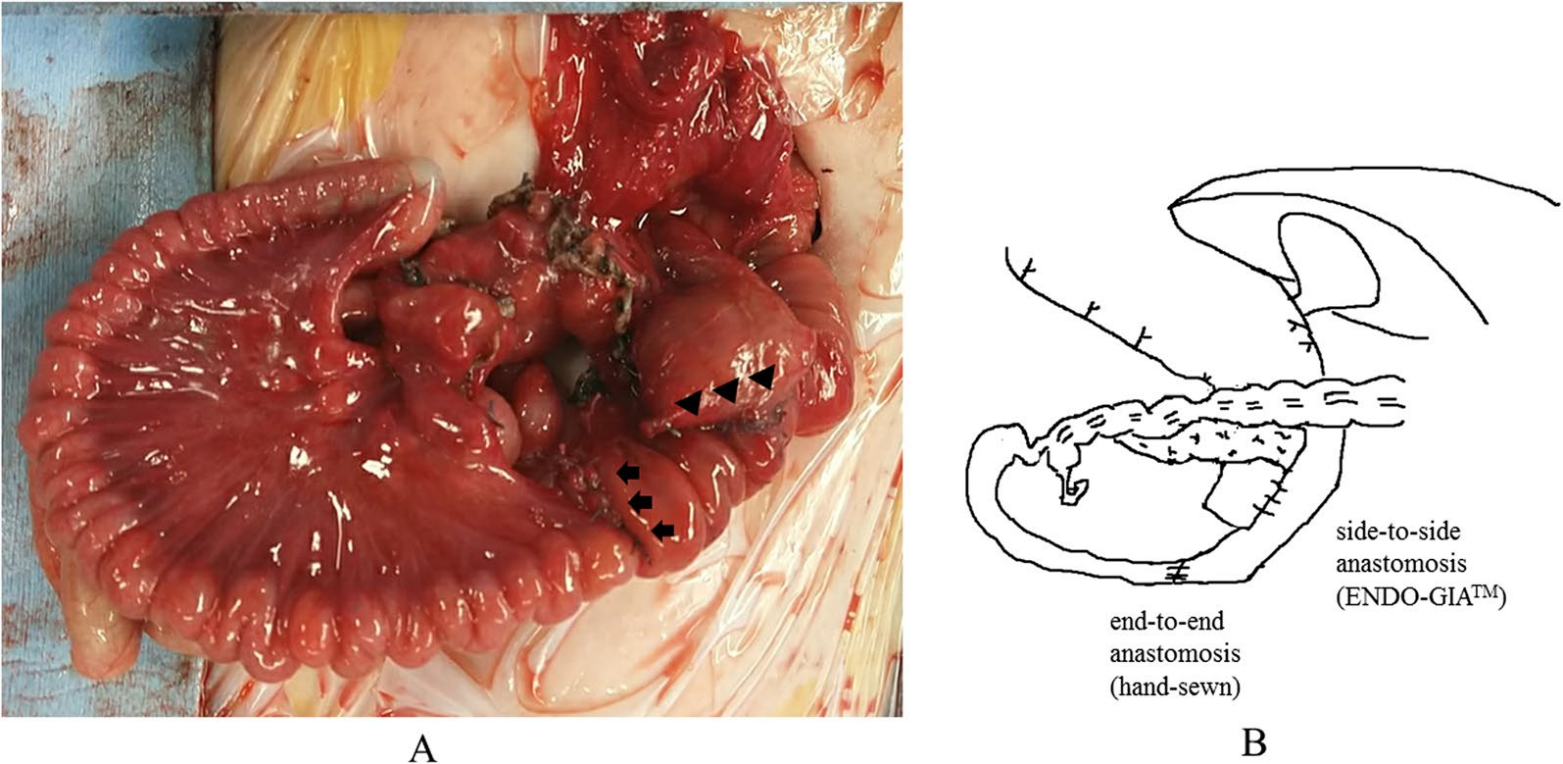
Findings of reconstruction. **A **Functional side-to-side anastomosis between the duplicated jejunum and true lumen of the jejunum was achieved with the Endo-GIA™ camel load (Medtronic, Minneapolis, MN, USA) (arrow head), and end-to-back anastomosis between the jejunum and ileum was achieved with absorbable suture (arrow). **B** Schema of reconstruction.

**Fig. 4:**
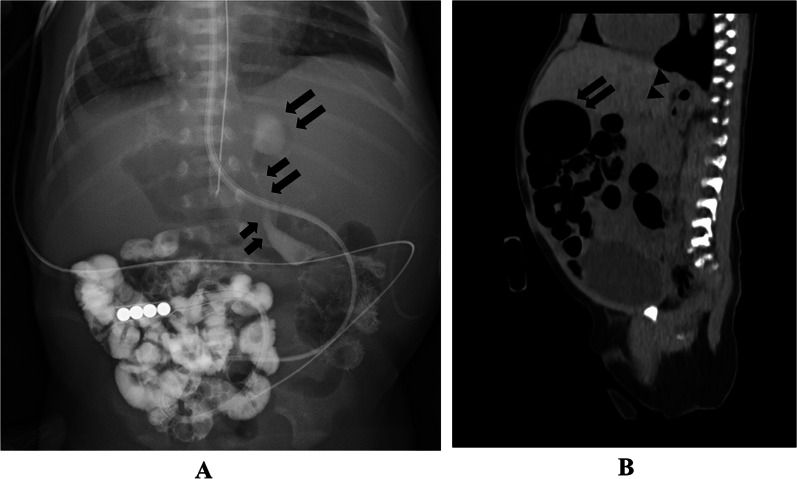
Postoperative imaging studies. **A** Postoperative gastrointestinal film showed a contrast agent pathway from the oral side of the side-to-side anastomosis to the dorsal area of the stomach, which was considered as remaining duplication (arrow). A 5-Fr tube was placed near the distal area of the anastomosis. **B** An abdominal computed tomogram revealed a luminal structure on the stomach’s dorsal area, which was compatible with the duplicated alimentary tract (arrow head). The stomach is located under the liver (arrow)

A 0-day-old female baby was referred to our department due to abdominal distention. During the prenatal stage, the baby was diagnosed with intestinal atresia based on the intestinal loop dilatation findings found via maternal ultrasonography. She was delivered vaginally at full term weighing 3.5 kg. X-ray examination revealed dextrocardia, left-sided liver, and shifting of the nasogastric tube to the right side (Fig. 1A). Abdominal ultrasonography test revealed multiple dilated intestinal loop and situs inversus (Fig. 1B). The baby was diagnosed as having intestinal atresia with situs inversus and underwent emergency exploratory laparotomy after adequate resuscitation on the day of birth. A transverse incision on the upper abdomen was performed, revealing completely reversed left and right abdominal organs. The origin of the jejunum was on the left side accompanied with tubular intestinal duplication. The origin of the duplicated intestine was at the dorsal area of the pancreatic head. The tubular intestinal duplication was on the mesenteric side with a length of 40 cm. There were two points of type Шa ileal atresia at the intervals of 5 cm each. The distal side of the ileum and the entire colon was collapsed **(**Fig. 2A, B). Initially, we tried to resect the duplicated intestine with the accompanying jejunum. However, the proximal end point of the duplicated intestine was on the dorsal area of the pancreatic head and was not dissected completely even after Kocher’s maneuver. In addition, it was unclear whether the ampulla of Vater was on the true lumen of the jejunum or duplicated intestine. Therefore, the duplicated intestine with the length of 3 cm was spared and anastomosed with the true lumen of the jejunum, and the residual duplicated intestine with the accompanying jejunum was resected. The duplicated intestine was transected at 3 cm from the lower border of the pancreatic head, and the true jejunum was transected at 5 cm from the origin. The absence of the ampulla of Vater in the observation range of both lumens was confirmed. Small holes were made on the each wall of the duplicated intestine and true jejunum, and functional-side-to-side anastomosis was achieved with a 45-mm Endo-GIA™ camel load (Medtronic, Minneapolis, MN, USA). The insertion point was closed with a 5–0 absorbable suture. The ileum was transected at the distal side of the atresia point, and the mesenteric vessels were clamped and resected. The end-to-end anastomosis was performed with a 5–0 absorbable suture. A 5-Fr tube was passed through before suture completion as a transanastomotic tube (Fig. 3A, B). The abdominal cavity was irrigated, and a drain was inserted into the Douglas’s pouch via the right lateral region; then, the wound was closed. The patient was extubated on day 6 postoperation. Gastrointestinal series on day 11 postoperation revealed no contrast agent leakage; however, a contrast agent pathway from the oral side of the side-to-side anastomosis to the stomach’s dorsal area was observed (Fig. 4A). In addition, an abdominal computed tomogram revealed a luminal structure on the stomach’s dorsal area, which was compatible with the duplicated alimentary tract (Fig. 4B). It was speculated that the oral end of duplication is the stomach and the contrast agent flew into this remaining area. Enteral nutrition was initiated and established safely, and the patient was discharged at 2 months postoperatively. She has been received H_2_ blocker, because there has been a concern about gastrointestinal hemorrhage caused by ectopic stomach mucosa in the residual duplicated tract. She has been under periodical medical surveillance without gastrointestinal complications such as inflammation or hemorrhage and will undergo resection of residual duplication.

## Discussion

The etiology of intestinal atresia is considered as intrauterine mesenteric vascular accidents, including compression, twisting, or embolization of fetal mesenteric vessels [1]. Mesenteric vascular accidents during the antenatal period result in short bowel, intestinal stenosis, intestinal atresia, and enteric duplication, depending on its severity [3]. Duplication may occur during the vascular accidents, followed by necrosis of the affected intestine and regeneration of the intestinal components with recovery of blood supply from the conterminous mesentery. Case reports on intestinal duplication associated with intestinal atresia are scarce [4–6]. In our case, isolated ileal atresia was found at the point of the anal end of the duplicated intestine. Therefore, the aforementioned mechanism is considered the cause of the coexistence of intestinal duplication and atresia in our case.

The radical treatment of the alimentary tract duplication is complete surgical resection; a deliberate decision is necessary in cases involving the duodenum. In some duodenal duplication cases, the possibility of the ampulla of Vater being involved in the duplication should be taken into consideration. In a previous case series of pediatric duodenal duplication, the biliary tree was not involved in the duplicated duodenum in four out of six cases, whereas in the other two cases, the ampulla of Vater was involved in the duplicated duodenum [7]. In the latter two cases with involvement of the ampulla of Vater, complete surgical resection of the common wall was adopted in one case, whereas, in another case, only an incomplete surgical resection of the common wall was possible, because the opening of the papilla was covered with the duplicated wall. It is necessary to identify whether the ampulla of Vater is located on the true or false lumen of the duodenum.

In our case, whether the ampulla of Vater is on the duplicated duodenum or true lumen of the duodenum at the time of the operation was unclear. Simple transection could result in the disruption of the passage of the biliary and pancreatic juices through the intestine if the ampulla of Vater had been on the duplicated lumen. We considered to perform an intraoperative cholangiography via the gall bladder to elucidate this issue. However, if the ampulla of Vater was revealed to be on the duplicated lumen, complete resection was impracticably difficult without reconstruction of outflow tract of biliary and pancreatic juices. Although pancreaticoduodenectomy had been considered as one of solutions in theory, it was also considered to be dangerous and excessive burden for the present neonatal case. Even though the ampulla of Vater was revealed to be on the true lumen, it was difficult to resect the duplicated tract completely, because the proximal end point of the duplicated tract was extended to the dorsal side of the pancreatic head in the retroperitoneal part. Stripping of the mucosa, as one of the alternative procedures, was difficult to perform because the extent of the duplication was unclear. Thus far, whether the ampulla of Vater was on the true or duplicated lumen, it was extremely difficult to resect the duplicated tract completely at the surgery. Therefore, we decided to spare the jejunum’s proximal part without intraoperative cholangiography. In a previous review involving 53 pediatric duodenal duplication cases, the duplication in 11 cases (21%) was connected to the pancreaticobiliary ducts [8]. Pancreatitis is among the frequently seen complications due to different etiologies, including transient mobility related to duodenal obstruction of the major papilla outflow, compression of the pancreatic duct or biliary tree by a large cyst, or obstruction of the pancreatic duct by migrating biliary sludge or small stones [9–11]. Our patient had no history of pancreatitis during the 1-year periodical medical surveillance. Previously, some adult cases with carcinogenesis of alimentary tract duplication were reported [12, 13]. Although the mechanism of malignant transformation of alimentary tract duplication and the duration for development of malignancies from the duplication have not been clearly identified, leaving the duplication for long period is unfavorable in the present case from this point of view. Therefore, we plan for resection of residual duplication,

We selected functional side-to-side anastomosis between the separated and duplicated jejunum using Endo-GIA™. Conventionally, stapled anastomosis had been adopted in gastrointestinal surgery in the adult population. The recent development of instruments with a smaller width and staplers with a smaller depth during the endoscopic surgical era allowed the application of stapled anastomosis to the pediatric population. In a previous randomized controlled study comparing hand-sewn anastomosis with stapled anastomosis in neonatal surgery, no significant differences in the anastomotic stricture, leakage, and other complications were found [14]. Moreover, when the lumen diameter was < 1.0 cm, stapled anastomosis was not applicable; otherwise, stapled anastomosis could be performed safely. In our case, we preserved the communication between the duplicated and true lumen of the duodenum after transection and separation to ensure the passage of biliary and pancreatic juices. Initially, we intended to perform hand-sewn anastomosis; however, we had to save time and sufficient anastomotic diameter was necessary. Thus, we selected stapled anastomosis with Endo-GIA™. The tissue thickness after compression with the camel road of Endo-GIA™ was 0.88–1.8 mm, which was appropriate for the patient’s intestinal anastomosis. Although the reports of the use of stapled anastomosis in neonates are scarce, its reported advantages include shorter operative time without an increased risk for postoperative complications including anastomotic leakage, stricture, and bleeding [14–16]. In our case, the stapled side-to-side anastomosis of the duplicated jejunum saved time and preserved the duplicated jejunum to allow passage of biliary and pancreatic juices.

## Conclusions

In our case, identifying whether the ampulla of Vater was on the duplicated duodenum or not during the surgery was difficult, leading us to spare the duplicated duodenum. Stapled side-to-side anastomosis between the duplicated duodenum and true tract was time saving and secured a sufficient anastomotic diameter in our case.

## Data Availability

The datasets supporting the conclusions of this article are included within the article.
